# Dopamine D_2_ receptors and the circadian clock reciprocally mediate antipsychotic drug-induced metabolic disturbances

**DOI:** 10.1038/s41537-017-0018-4

**Published:** 2017-04-10

**Authors:** Zachary Freyberg, Michael J. McCarthy

**Affiliations:** 1 Department of Psychiatry, University of Pittsburgh, Pittsburgh, PA 15213 USA; 2 Department of Cell Biology, University of Pittsburgh, Pittsburgh, PA 15213 USA; 3 Psychiatry Service, Veterans Affairs San Diego Healthcare System, San Diego, CA 92161 USA; 4 Department of Psychiatry, University of California San Diego, La Jolla, CA 92093 USA

## Abstract

Antipsychotic drugs are widely prescribed medications, used for numerous psychiatric illnesses. However, antipsychotic drugs cause serious metabolic side effects that can lead to substantial weight gain and increased risk for type 2 diabetes. While individual drugs differ, all antipsychotic drugs may cause these important side effects to varying degrees. Given that the single unifying property shared by these medications is blockade of dopamine D_2_ and D_3_ receptors, these receptors likely play a role in antipsychotic drug-induced metabolic side effects. Dopamine D_2_ and dopamine D_3_ receptors are expressed in brain regions critical for metabolic regulation and appetite. Surprisingly, these receptors are also expressed peripherally in insulin-secreting pancreatic beta cells. By inhibiting glucose-stimulated insulin secretion, dopamine D_2_ and dopamine D_3_ receptors are important mediators of pancreatic insulin release. Crucially, antipsychotic drugs disrupt this peripheral metabolic regulatory mechanism. At the same time, disruptions to circadian timing have been increasingly recognized as a risk factor for metabolic disturbance. Reciprocal dopamine and circadian signaling is important for the timing of appetitive/feeding behaviors and insulin release, thereby coordinating cell metabolism with caloric intake. In particular, circadian regulation of dopamine D_2_ receptor/dopamine D_3_ receptor signaling may play a critical role in metabolism. Therefore, we propose that antipsychotic drugs’ blockade of dopamine D_2_ receptor and dopamine D_3_ receptors in pancreatic beta cells, hypothalamus, and striatum disrupts the cellular timing mechanisms that regulate metabolism. Ultimately, understanding the relationships between the dopamine system and circadian clocks may yield critical new biological insights into mechanisms of antipsychotic drug action, which can then be applied into clinical practice.

## Introduction

Antipsychotic drugs (APDs) are commonly prescribed across a range of psychiatric illnesses including schizophrenia, major depression, bipolar disorder (BD), aggressive behaviors, dementia, anxiety, and post-traumatic stress disorder (PTSD). Consequently, APDs are some of the most widely prescribed medications in the U.S. with 1.2% of the population filling APD prescriptions.^1^ Moreover, public spending on APDs has increased ~ 5-fold in under a decade to $4.6 billion annually.^2^ However, despite their many uses, APDs have significant metabolic side effects, causing weight gain, increased risk of type 2 diabetes (T2D), cardiovascular disease and metabolic disturbances characterized by abdominal obesity, glucose intolerance, insulin resistance, hypertension, and dyslipidemia. While second-generation APDs like olanzapine are well-known to cause substantial weight gain,^3^ even first-generation APDs such as haloperidol, commonly considered safer from a metabolic perspective, may produce significant metabolic disturbances.^4^ Indeed, a recent multi-center study, the European First Episode Schizophrenia Trial concluded that metabolic disturbances and weight gain are associated with many APDs—both first and second generation.^4^ These metabolic side effects have led to high rates of treatment discontinuation, requirements for careful monitoring, and worse clinical outcomes, putting considerable strain on health-care systems. Consequently, psychiatric patients treated with these medications have markedly elevated rates of mortality across the lifespan, even after correcting for elevated rates of suicide associated with the disorders.^5^ Much of the mortality risk is attributed to cardiovascular disease, obesity, and metabolic disease.^6, 7^ Thus, developing a better understanding of the mechanism(s) of APD-induced metabolic abnormalities may ultimately lead to novel clinical interventions, leading to fewer metabolic side effects and improved treatment compliance. However, to date, the mechanisms for APD-induced metabolic abnormalities remain poorly understood. This is likely due to complexity in the system, with the convergence of at least three of the biological pathways involved in regulating metabolism and weight homeostasis including: (1) insulin control of glucose metabolism in the pancreas (2) central nervous system (CNS) regulation of mechanisms involved in appetite, satiety, and feeding behaviors, and (3) the coordination among these factors to ensure optimal timing of metabolic functions across a range of circumstances (e.g., circadian rhythms in sleep/activity).

## APDs target central and peripheral dopamine receptors

Although multiple cellular targets for APDs, including histamine and serotonin receptors^8^ have been identified, all clinically effective APDs act through dopamine D_2_ (D2R) and D_3_ (D3R) receptors, suggesting a critical role for dopamine in promoting not only the therapeutic actions of these drugs, but possibly also in causing their metabolic side effects. APDs achieve their effects as both antagonists and inverse agonists of D2R and D3R, able to inhibit cellular signaling even in the absence of endogenous dopamine.^9, 10^ In the CNS, there exist distinct populations of tyrosine hydroxylase (TH)-expressing, dopaminergic neurons. In the midbrain, these neurons include cells in the ventral tegmentum (VTA) that project to the ventral striatum and frontal cortical regions implicated in modulating reward,^11^ and a distinct population in the substantia nigra that project to the dorsal striatum to control motor activity.^12^ In the hypothalamus, dopaminergic neurons in the arcuate nucleus (ARC) project locally to pro-opiomelanocrotin (POMC)-containing neurons that play a critical role in central control of glucose and energy homeostasis, in part through regulation of appetite and feeding.^13, 14^ Therefore, D2R and D3R in these striatal and hypothalamic dopamine circuits mediate appetite and feeding behaviors by stimulating the neural pathways that govern motivation, motor activity, and reward.^15^ The distribution of D2R and D3R in the striatum and ARC suggests that APDs could induce metabolic dysfunction by interfering with the dopaminergic mechanisms involved in feeding and satiety in the CNS.^16^

**Fig. 1:**
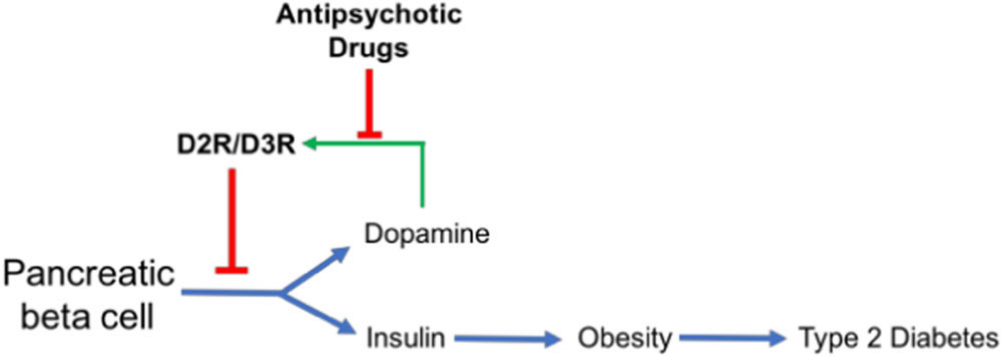
Model for dopamine D2/3 receptor-mediated regulation of insulin release in the pancreatic beta cell. Dopamine acts through a dopamine D_2_/D_3_ receptor (D2/D3R)-mediated autocrine negative-feedback mechanism to inhibit further insulin release. Left unchecked following APD treatment, chronic insulin release may promote adipogenesis, weight gain, insulin resistance, and ultimately type 2 diabetes. The figure was adapted from Rubí and Maechler [*Endocrinology* 151(12): 5570–5581, 2010] with permission from Endocrinology and HighWire Press via Copyright Clearance Center

Peripheral dopamine outside the CNS may also be playing important roles in metabolic regulation. Surprisingly, D2R and D3R are also expressed in insulin-secreting pancreatic beta cells.^17^ These cells possess the machinery for both dopamine and insulin biosynthesis. We and colleagues showed that D2R/D3R can regulate both insulin and dopamine release directly in pancreatic beta cells from humans and rodents independently of CNS control.^17–20^ Specifically, we found that pancreatic dopamine inhibits glucose-stimulated insulin secretion (GSIS) via beta cell D2R/D3R, through an autocrine/paracrine mechanism.^18, 20^ Conversely, APD blockade of D2R/D3R significantly enhances GSIS.^18^ Importantly, this APD-induced increase in insulin secretion reproduces aspects of the chronic hyperinsulinemia found in T2D^21^ (Fig. 1). Rodent models of APD-administration recapitulate these findings, showing weight gain and peripheral insulin resistance.^22^ These studies point to direct action of APDs on peripheral dopamine targets, including insulin-secreting pancreatic beta cells as potentially important contributors to APD-induced metabolic dysfunction.^20^

Many metabolic systems are rhythmic, under the control of central and peripheral circadian clocks. Therefore, in addition to the role of dopamine signaling, circadian rhythms may also significantly contribute to in APD-induced metabolic disturbances.^23^

## Circadian rhythms are determined by the molecular clock

**Fig. 2:**
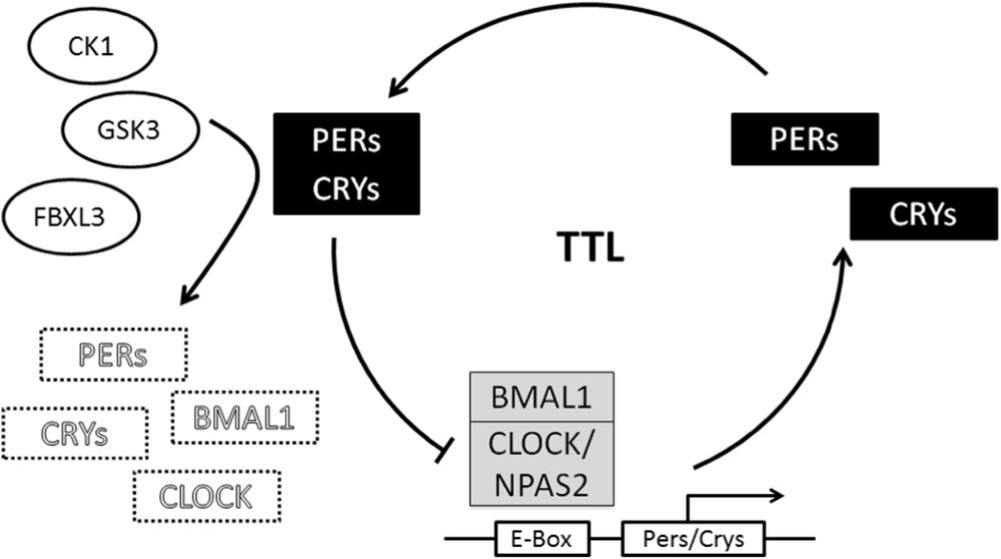
The circadian transcription/translation loop is comprised of rhythmically expressed “clock genes”. Circadian protein complexes containing BMAL1 drive expression of rhythmic genes including period (PER) and cryptochrome (CRY) through E-box DNA elements in promoters. PER/CRY negatively regulate their own expression. As PER/CRY degrade over several hours, BMAL1 activity increases and the cycle repeats. Time of onset (phase), duration (period), and strength (amplitude) of the cycle is regulated by multiple signaling pathway inputs. The figure was adapted from Landgraf *et al*. [*Behavioral Neuroscience* 128(3): 344–359, 2014] with permission from the American Psychological Association

Most mammals show endogenous 24-h physiological rhythms, termed circadian rhythms, that synchronize the activity of organ systems with each other and the environment. The biological basis of circadian rhythms in mammals, including humans, is now well understood. The master timekeeper is located in the CNS, within the suprachiasmatic nucleus (SCN) of the hypothalamus and responds to light input from specialized photoreceptors in the retina as the primary signal that determines circadian phase.^24^ Circadian clocks are cell autonomous and ubiquitous across tissues, meaning that cells across a wide variety of brain regions outside the SCN,^25–27^ and peripheral organs have the ability to maintain daily rhythms in gene expression and physiology.^28^ A transcriptional/translational feedback loop made up of ~ 20 “core clock genes” exists to maintain essential functions underlying cellular circadian rhythms (Fig. 2). At the center of this loop, the proteins CLOCK and BMAL1 interact to form a heterodimeric transcriptional activator. Under some circumstances, another circadian transcription factor, NPAS2 can substitute for CLOCK within this dimer.^29^ The BMAL1 complex binds to E-box promoter elements, driving the expression of CRY1/2, and PER1/2/3 proteins, transcriptional repressors that inhibit their own expression to sustain circadian oscillators with a period length of ~ 24-h (Fig. 2). Additional negative feedback loops comprised of REV-ERB-α/β proteins play important supporting roles, acting through distinct promoter elements to sustain rhythmic expression of genes across the genome.^30^ Post-translational, bioenergetic and epigenetic factors can also contribute to the maintenance of rhythms.^31^ In particular, multiple signal transduction pathways (e.g., cyclic adenosine monophosphates, cAMP) and activated protein kinases such as extracellular signal-regulated kinase (ERK) and p38 serve as clock inputs,^32^ linking neurotransmitter systems (e.g., dopamine) to the circadian clock through post-translational modification of clock proteins and/or modulation of transcription factors that regulate clock gene promoter elements (Fig. 2).

## Peripheral circadian rhythms and metabolism

Food is a powerful time-keeping cue that can direct biological rhythms. In peripheral organs such as the liver, food may be a more important time cue than light under restricted feeding conditions.^33^ Under healthy physiological conditions, insulin secretion, target tissue insulin sensitivity, glucose metabolism, leptin signaling, and lipid metabolism follow circadian rhythms. These rhythms generally favor anabolic activity during the daily active period when feeding occurs, and catabolic activity at rest.^23^ Accordingly, circadian clocks in peripheral organs including adipocytes,^34, 35^ liver,^36^ and pancreas^37, 38^ regulate these metabolically relevant rhythms, helping the body adjust for the cycles of prolonged fasting associated with sleep or during periods of caloric restriction. Rhythms also mediate glucose utilization in insulin-sensitive target tissues such as skeletal muscle.^39^

Increasing evidence suggests that timing is critical for maintaining metabolic homeostasis.^40–43^ When temporal control over diurnal and/or circadian rhythms is lost, it may lead to an inability to process glucose effectively, and ultimately to the development of metabolic disturbances.^40, 43^ Indeed, in mice fed an unrestricted high-fat diet across the day, this loss of diurnal feeding rhythms resulted in profound metabolic disturbances (e.g., weight gain, hyperinsulinemia, hepatic steatosis, and elevated markers of inflammation).^44^ In contrast, when feeding was time restricted to the active cycle, the mice were protected from the adverse metabolic changes, despite consuming identical numbers of calories.^44^ These data suggest that besides the caloric and nutrient composition of the diets, optimal control of timing for food consumption may also contribute significantly in determining metabolic outcomes.

Pancreatic release of insulin follows a circadian rhythm, peaking during the day in response to feeding and dropping at night during sleep.^41^ Although influenced by food intake, these rhythms operate independently and can anticipate regular changes in feeding behavior and/or activity.^41^ Recent evidence shows that cellular timing is critical for proper insulin release, and that a fundamental property of insulin-secreting beta cells is their capacity to synchronize, and function as rhythmic pacemakers.^38, 40, 45^ Interference with these beta cell rhythms may cause conditions that resemble T2D.^45^ Whole animal disruption of the circadian clock using CLOCKΔ19 mutants and BMAL1 knockouts leads to impaired glucose tolerance, deficits in insulin secretion, and excessive fat deposition,^46, 47^ indicating a major contribution of the circadian system to metabolic homeostasis. To disentangle the contributions of specific organ systems to these phenomena, more precise pancreatic beta cell-specific CLOCKΔ19 mutants and BMAL1 knockouts revealed that these beta cell clocks are directly responsible for the observed impairments in hyperglycemia, glucose tolerance, and GSIS.^37^ Moreover, it has been discovered that pancreatic clocks regulate the transcription of many of the genes responsible for the rhythmic release of insulin throughout the day,^38^ and that an intact circadian pacemaker intrinsic to beta cells is critical for regulating GSIS.^48^

Consistent with the animal data, disruptions in circadian cycles are a major risk factor for the development of obesity and metabolic health problems in humans.^49^ Epidemiological,^50^ observational^51^ and controlled laboratory studies^52^ have repeatedly demonstrated that disruption of circadian rhythms causes insulin resistance, elevated blood glucose and may ultimately lead to obesity and diabetes.^53^ Sleep loss has also been associated with metabolic disturbances.^54, 55^ These sleep and circadian disruptions are particularly problematic among populations engaged in shift work and/or frequent long haul travel—activities which disrupt rhythms, and, therefore, increase metabolic risk.^23, 56, 57^ Off-label prescription of APDs for insomnia has further exacerbated the preexisting metabolic risks stemming from job-related circadian disruptions within these populations,^58^ making this a particularly relevant public health issue. While the relationship of metabolic dysfunction to sleep is complex, circadian disruption may be an important factor. Understanding the biological mechanisms underlying these metabolic disturbances may shed light on several psychiatric illnesses where circadian disruptions are salient pathophysiological features.^59^

## CNS circadian rhythms and metabolism

Disturbances in the dopamine and circadian systems have been implicated in the pathophysiology of BD and schizophrenia. Both disorders are associated with disruptions of sleep and activity, as well as obesity, independent of APD exposure.^59, 60^ Therefore, even in the absence of APD treatment, changes in circadian phase or loss of rhythms in the dopamine systems may contribute to metabolic problems in the mentally ill.^59^ Exposure to APDs may further worsen these metabolic effects, even as they treat psychiatric symptoms.^61^ Nevertheless, the precise mechanisms by which these systems contribute to metabolic dysfunction remain poorly understood.

There is evidence that CNS and peripheral circadian systems work closely with one another to maintain metabolic homeostasis.^61, 62^ The periphery communicates information to the CNS in a rhythmic manner. For example, adipocytes rhythmically release leptin, a neuroactive peptide that regulates satiety through its actions centrally in the ARC.^35^ When leptin rhythms are disrupted in mutant mice lacking both Per1 and Per2, or both Cry1 and Cry2, animals show profound, metabolic phenotypes (weight gain and weight loss, respectively). These changes are mediated by rhythmic behavior and feeding^35^ and highlight the role of the circadian system as a coordinator of physiology across peripheral organs and the CNS.

Metabolic homeostasis requires a balance between energy intake and expenditure.^63, 64^ Feeding behavior (intake) and locomotion (expenditure) have both motivational and motor aspects that are involved in striking this balance, and are controlled by two complementary dopamine systems in the striatum.^65^ Rhythmic dopamine signals that originate in the VTA and project to the ventral striatum [nucleus accumbens (NAc)] regulate the reward pathways underlying motivation, food craving, and anticipation.^66^ In parallel, dopamine neurons in the substantia nigra project to a distinct set of dorsal striatal neurons to regulate aspects of motor control, including locomotion. Similar to feeding, locomotor behaviors are rhythmic under constant conditions. Elimination of neurons projecting to the striatum using the dopamine-specific toxin 6-hydroxydopamine (6-OHDA) rendered locomotor activity arrhythmic, and disrupted the rhythmic expression of PER2 in the dorsal striatum, but not in the SCN.^67^

The ARC region of the hypothalamus also includes a distinct population of dopaminergic neurons that regulate satiety and appetitive behaviors.^26, 68^ The ARC exhibits strong rhythms in gene expression that shift phase in response to food deprivation.^69^ Furthermore, optogenetic stimulation of ARC dopaminergic neurons and D2R signaling in the ARC similarly regulates appetite and feeding behavior.^13^ Dopamine signaling also affects the rhythmic anticipatory behaviors that determine feeding time in mice.^70^ A significant component of this signaling is D2R/D3R-dependent, where receptor action affects the time of onset in feeding behaviors.^71^

Both D2R/D3R signaling and circadian pathways have been implicated in the temporal coordination of satiety and weight gain.^72–75^ Interestingly, repeated stimulation of the dopamine system by amphetamine administration is sufficient to sustain rhythmic anticipatory behaviors even when the SCN-driven master clock is impaired.^76^ This anticipation may reflect the activity of ultradian rhythm (i.e., shorter than 1 day) that are governed by the midbrain dopamine systems and striatal neurons. These rhythms typically run in 2–6 h cycles that harmonize with, and support the 24-h circadian rhythms. However, desynchronization of ultradian and circadian rhythms by excessive dopaminergic tone can lead to interference in rhythmic behaviors.^77^ Therefore, disruptions of the CNS dopamine and circadian systems may change reward systems in the NAc, alter satiety set points, and contribute to overeating and obesity.^73, 75, 78^ Overall, this evidence suggests that CNS dopamine, D2R and D3R play critical roles in mediating rhythmic feeding behaviors and satiety, and that the loss of circadian regulation may result in clinically relevant disturbances in maintaining weight control.

## Dopamine receptors are localized to rhythmic brain areas

The bidirectional connections between the dopamine system and the circadian clock are determined in large part by D2R and D3R expression, which is widespread across brain regions that support circadian rhythms (Table 1). In particular, D2R/D3R expression is enriched in the striatum (NAc and caudate/putamen) and midbrain (substantia nigra, VTA). Expression of D2R (but not D3R) has been described in the ARC. Strikingly, there is no expression of D2R/D3R in the SCN. Correspondingly, there is a relative resistance of SCN rhythms to D2R/D3R signaling.^79^ Instead, dopamine D_1_ receptors (D1R) are the primary dopamine receptor in the SCN.^80^ These findings imply that there are dopaminergic mechanisms that distinguish SCN rhythms from the D2R/D3R-mediated mechanisms in the striatum and elsewhere in the hypothalamus.

**Table 1:** D2R and D3R expression profiles in brain regions that exhibit 24-h clock gene oscillations

Brain Structure	D2R	D3R	Sustains rhythms	Rhythmic process implicated	References
SCN	−	−	+++	Master clock	Landgraf *et al*.^27^ Bouthenet *et al*.^98^
ARC	+	−	++	Feeding behavior	Guilding *et al*.^26^ Romero-Fernandez *et al*.^68^, Bouthenet *et al*.^98^
NAc	+++	+++	++	Reward/motivation	Landgraf *et al*.^27^
CPU	+++	+++	++	Locomotion	Natsubori *et al*.^25^
VTA	+++	+/-	+	Reward/motivation	Landgraf *et al*.^27^ Gurevich *et al*.^99^
SN	+++	+	++	Locomotion	Natsubori *et al*.^25^ Gurevich *et al*.^99^

Brain regions involved in dopamine signaling support circadian rhythms. Shown above is D2R and D3R expression in the SCN, and selected brain regions outside the SCN that maintain circadian rhythms. D2R/D3R expression reflects gene expression data and/or immunocytochemistry as reported in the associated reference. Expression is marked high (+++), medium (++), low (+) or absent (−). Expression of D3R in VTA differs in human and rat. Ability to sustain rhythms was defined by results of bioluminescent reporter assays conducted in brain slices ex vivo. Rhythm amplitudes are coded as strong (+++), moderate (++) or weak (+). Measures of in vivo rhythms determined by neurochemical, electrophysiological, or behavioral assays are not addressed, but are present in many cases. Arcuate nucleus (*ARC*), caudate/putamen (*CPU*), nucleus accumbens (*NAc*), suprachiasmatic nucleus (*SCN*), substantia nigra (*SN*), ventral tegmental area (*VTA*)

## Reciprocal links between dopamine signaling and the circadian clock

Dopamine neurotransmission in the brain is rhythmic and tightly regulated by the circadian clock.^81^ In the striatum, microdialysis experiments in animals indicate that dopamine release from presynaptic neurons undergoes rhythmic oscillations across the day.^82^ Similar work by multiple groups indicates that the regulatory sites underlying these oscillations may be distributed across multiple nodes in the dopamine system. In dopamine synthetic pathways of mouse midbrain neurons, circadian regulators CLOCK^83^ and REV-ERB^84^ regulate the expression of TH (the rate limiting step in dopamine biosynthesis). Moreover, in the striatum, D3R is expressed rhythmically under the control of the circadian regulator, NPAS2.^66^ Importantly, disrupting these circadian rhythms genetically or pharmacologically with APDs alters D3R expression and leads to behavioral disturbances affecting reward pathways.^66^ Finally, critical negative regulators of dopamine signaling are also rhythmically expressed throughout the day including the dopamine transporter^82^ as well as monoamine oxidase A.^85^

There is growing evidence showing that D2Rs and D3Rs are integrators of multiple signaling pathways including those associated with APD-induced metabolic abnormalities.^61^ Accordingly, the dopamine and circadian systems likely converge through their joint actions at dopamine receptors. As G-protein coupled receptors, both D2R and D3R have the capability of acting through both G-protein-dependent and -independent pathways.^86^ In the case of the G-protein-dependent pathways, D2R and D3R signaling is coupled to the downstream actions of stimulatory or inhibitory G_α_ subunits, which modulate intracellular levels of cyclic AMP (cAMP).^87^ G-protein independent/arrestin-dependent signaling modulates other classes of signaling molecules like the AKT kinase and intracellular calcium.^86^ These components of the dopamine signaling network provide inputs into the circadian clock. Indeed, we have shown previously that D1R modulate circadian period length in SCN slices from PER2::LUC mice,^88^ while others demonstrated that D2Rs and D3Rs affect modulation of the CLOCK/BMAL1 dimerization in the retina.^89^ Moreover, the same dopamine receptors determine the time of peak PER2 expression in the striatum.^79^

The extensive interplay between dopamine and circadian systems both in the CNS and periphery increases the probability that feeding behaviors and activity are coordinated. This ensures that dopamine-driven hunger and feeding behaviors occur at times of caloric need, to efficiently balance energy intake and expenditures. Misalignment of circadian rhythms results in metabolic changes similar to those induced by APDs, including changes in satiety, increased blood glucose and lipid levels, and the development of obesity and T2D.^56^ Taken together, temporal and reciprocal coordination between metabolic and dopamine systems may be critical to maintaining optimal control of insulin and blood glucose.

## Clinical implications of reciprocal dopamine and circadian signaling

One of the most studied clinical models of dopamine signaling is Parkinson’s disease (PD). PD is characterized by a reduction of dopamine signaling in the dorsal striatum, leading to deficits in locomotion and cognition. Interestingly, in addition to these behavioral abnormalities, early clinical observations indicated that these patients also commonly exhibit disturbances in glucose homeostasis.^90^ Indeed, in PD patients, administration of L-DOPA, a dopamine precursor, acutely increased blood glucose.^91^ These data suggest that dopamine signaling may have physiological relevance beyond the CNS, including a role in glucose regulation and that disruption of this signaling may lead to pathological metabolic states. Emerging data from human clinical trials support a circadian and dopamine interaction in regulating, leptin, insulin, peripheral blood glucose and metabolism. Specifically, the D2R/D3R agonist bromocriptine has shown efficacy in T2D, and may act in part through a circadian mechanism.^92^ Bromocriptine has been shown in placebo-controlled studies to modulate circadian rhythms in obese subjects, reducing the peak amplitude and mean levels of rhythmic insulin levels across a 24-h cycle.^93^ Similar results have been reported for the effects of bromocriptine on rhythmic leptin levels, whereby bromocriptine treatment of obese subjects reduced the rhythm amplitude and mean levels of leptin over 24-h, and stimulated lipolysis.^94^ Since completion of these studies, bromocriptine has been approved by the FDA to treat T2D by restoring insulin sensitivity to target tissues. By elucidating the interactions between the dopamine and circadian systems, these bromocriptine studies may offer important preliminary clues to understand how APDs alter dopamine signaling and cause metabolic disease. However, considerably less is known about the effects of APDs at the convergence of dopamine and circadian pathways. There is a paucity of data to support the use of bromocriptine for the treatment of APD-induced metabolic disorders in psychiatric patients. The existing literature suggests that bromocriptine does not exacerbate psychosis in APD-treated patients,^95–97^ but larger studies to investigate the relative risks and benefits of dopaminergic agonists are clearly needed.

**Fig. 3:**
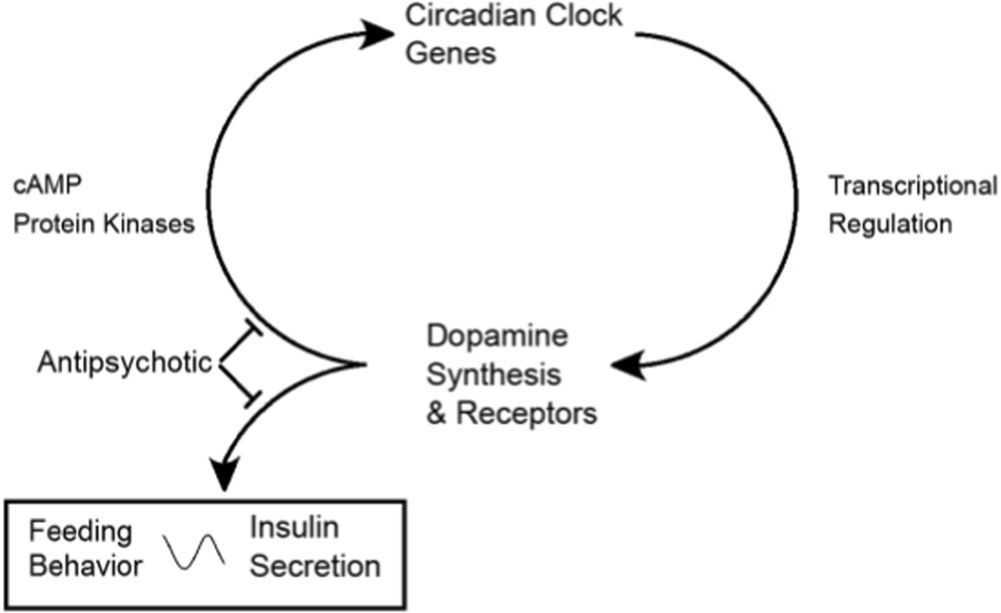
Bidirectional connections link circadian clock genes to dopamine. Rhythmic control of dopamine synthesis and signaling in pancreatic beta cells may be one mechanism by which insulin rhythms are maintained. In blocking dopamine signaling, APDs may interfere with (1) rhythmic insulin secretion and (2) dopamine receptor feedback to the beta cell circadian clock. The timing of feeding behaviors may influence rhythmic release of peripheral insulin

**Fig. 4:**
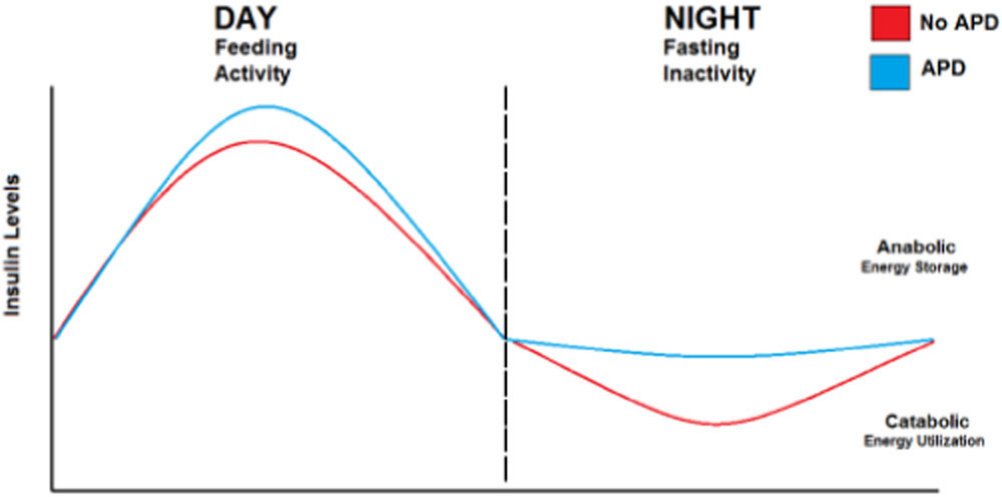
Model for antipsychotic drug disruption of circadian rhythms in insulin secretion. Insulin secretion follows a diurnal pattern with peaks during the day and lowest levels at night, corresponding to metabolic demands, periods of fasting and feeding behaviors in humans. By blocking dopamine D_2_ and D_3_ receptors, antipsychotic drugs (*APDs*) decrease the ability of dopamine receptors to negatively regulate insulin levels, raising overall insulin levels. At night time, during periods where catabolic states normally predominate, changes in insulin release by these drugs may be especially evident. Unique biological and pharmacological properties of different APDs (e.g., half-life) may alter the effects on insulin rhythms

The data suggest that APDs may interfere with the coordination between circadian and metabolic systems to affect clinical outcomes in patients treated with APDs (Fig. 3). As a corollary, this also implies that the timing of dopamine blockade by APDs may determine their effects on brain and peripheral rhythms. In particular, given that a common side effect of APDs is somnolence, these medications are often dosed at night to promote sleep. Since rhythmic insulin release is typically lowest at night, blockade of dopamine signaling by APDs during this time would be expected to cause inappropriately high insulin release. This could ultimately lead to the activation of anabolic pathways during a period when catabolic pathways should predominate, and promotes the weight gain and insulin resistance commonly observed during APD administration (Fig. 4).

We ultimately hypothesize that APD blockade of D2R and D3R disrupts circadian rhythms, disturbs the coordination of insulin release and feeding behaviors across the day in the CNS and pancreatic islet cells, and thereby induces metabolic dysfunction. This opens the door to several important questions: (1) Do APDs contribute to APD-induced metabolic disease by disrupting peripheral pancreatic circadian rhythms? Are these drug-induced disturbances dependent on pancreatic D2R/D3R? (2) By acting on D2R/D3Rs in the CNS, do APDs disturb circadian rhythms in brain regions that regulate feeding and activity to produce the changes in satiety and feeding that contribute to APD-induced obesity and metabolic disturbances? (3) What are the relative contributions of CNS and peripheral circadian D2R/D3R signaling to APD-induced disturbances to metabolism in vivo, and are there synergistic effects across CNS and peripheral mechanisms? (4) Do changes to the timing of administration alter the propensity of APDs to cause weight gain and metabolic dysfunction in humans?

## Future directions

Though much work has been done examining the dopamine and circadian systems individually, considerably more remains to be done in elucidating the interplay between these systems and its relevance to APD-induced metabolic disturbances. Given the importance of these two systems in the CNS and peripheral organs, it will be crucial to clarify their respective contributions both to behavioral and endocrine drivers of metabolic regulation. Understanding these factors will also shed considerable light on APD-induced metabolic disease. In the longer term, this knowledge may directly lead to the development of inexpensive and readily implemented treatment strategies to mitigate APDs’ metabolic side effects and reduce the morbidity and mortality from medication-associated T2D and cardiovascular disease. With fewer metabolic side effects, time-based interventions may also improve treatment compliance for disorders ranging from schizophrenia and BD to PTSD. Moreover, understanding interactions between dopamine and the circadian clock to regulate insulin release and feeding may also significantly contribute to our fundamental understanding of obesity and lead to novel treatments. Ultimately, further elucidating the mechanisms of APD-induced weight gain may also lead to better treatments for diabetes and obesity, which will directly impact the health of APD-treated patients.
